# Functional constipation masked as irritable bowel syndrome

**DOI:** 10.1186/s12876-020-01244-9

**Published:** 2020-04-06

**Authors:** Monica Tosto, Paola D’Andrea, Ignazio Salamone, Salvatore Pellegrino, Stefano Costa, Maria Cristina Lucanto, Socrate Pallio, Giuseppe Magazzu’, Stefano Guandalini

**Affiliations:** 1 Pediatric Gastroenterology and Cystic Fibrosis Unit, Policlinico G. Martino Hospital, University of Messina, Via Consolare Valeria 1, 98125 Messina, Italy; 2 Pharmacy Department, University of Bari, Bari, Italy; 3 Oncological Radiology Unit, Department of Biomedical and Dental Sciences and Morphofunctional Imaging, Policlinico G. Martino Hospital, University of Messina, Messina, Italy; 4 Digestive Diseases Endoscopy Unit, Policlinico G. Martino Hospital, University of Messina, Messina, Italy; 5 Adult and Pediatric Pathology Department G. Barresi, University of Messina, Messina, Italy; 6 Section of Pediatric Gastroenterology, Hepatology and Nutrition, University of Chicago, Chicago, USA

**Keywords:** Functional gastrointestinal disorders, Irritable bowel syndrome, Functional constipation, Occult constipation, Abdominal pain, Diarrhea

## Abstract

**Background:**

Rome IV criteria for functional gastrointestinal disorders state that children suspected of having Irritable Bowel Syndrome (IBS) with Constipation (IBS-C) should be preliminarily treated for constipation. We aimed at verifying if functional constipation may indeed lead to an erroneous diagnosis of IBS with diarrhea (IBS-D) or IBS with mixed pattern of diarrhea and constipation (IBS-M).

**Methods:**

We prospectively enrolled in an unblinded fashion 10 and 16 consecutive children referred to our center who met Rome IV criteria for a diagnosis of IBS-D and IBS-M, respectively. Patients who fulfilled criteria for suspect “occult constipation” were then given a bowel cleaning regimen with Polyethylene glycol 3350, re-evaluated at 2 months and followed up for at least 6 months. Sixteen additional patients with IBS with Constipation (IBS-C) referred in the same period served as control. The endpoints were: 1) a decrease of more than 50% in abdominal pain intensity and frequency scores; and 2) for patients with IBS-D and IBS-M: resolution of diarrhea.

**Results:**

The endpoints were met by 8 (80%) and 14 (87%) of the patients with IBS-D and IBS-M, respectively, with decrease of abdominal pain and resolution of “diarrhea”. The response was not significantly different from that observed in 15 (93%) of the IBS-C control group.

**Conclusion:**

Acknowledging the limitations of the small number of patients and of the uncontrolled nature of the study, we suggest that a possibly large number of patients labeled as IBS-D or IBS-M may actually simply present functional constipation and should be managed as such.

## Background

Functional gastrointestinal disorders (FGIDs) are common in children of all ages and cover a wide range of disorders associated with chronic, recurrent symptoms attributable to the gastrointestinal tract, which cannot be related to structural or biochemical abnormalities. FGIDs are diagnosed and classified according to symptom-based criteria, the so-called Rome criteria, of which the IV edition has been published [1, 2]. In 2018, Koppen et al. [3] systematically reviewed literature regarding the epidemiology of functional constipation (FC). The prevalence of constipation in the general pediatric population ranges widely from 0.5 to 32.2%. In addition, “occult constipation” should be taken into account. According to Gijsbers et al. [4], occult constipation is diagnosed in patients who do not fulfill the Rome criteria of constipation but had relief of symptoms with laxative treatment. Irritable Bowel Syndrome (IBS) accounts for 45% of cases of functional abdominal pain in children [5]. Abdominal pain-related IBS, even in absence of alarm signals [2], often gives rise to prolonged, unnecessary diagnostic testing and protracted attempts of treatment, leading only to more worry for the affected children and their parents, with major effects on direct and indirect costs for patients’ families and for healthcare systems. As noted by Saps et al. [6], the new Rome IV criteria introduced subcategories of previously existing diagnoses such as IBS with diarrhea, constipation, mixed (IBS-D, IBS-C and IBS-M, respectively) and unclassified. Previously, using the Rome III criteria for pediatric functional gastrointestinal disorders Giannetti et al. [7] had defined the prevalence, at diagnosis and at follow-up, of subtypes of irritable bowel syndrome in children, and, contrary to what found in adults, showed that IBS-D was more frequent in boys than in girls. Rome IV criteria state that in children with IBS-C the pain does not resolve with resolution of the constipation, as children in whom the pain resolves have functional constipation, not IBS. However, we speculate that in clinical practice, often, patients who receive a diagnosis of IBS-D and IBS-M may be indeed erroneously classified as such, as the apparent diarrhea could be secondary to overflow incontinence and the abdominal pain secondary to fecal retention [4]; hence the diagnostic approach should be first focused on ruling out FC.

Our aim was therefore to verify the hypothesis that a substantial portion of subjects diagnosed as having IBS-D or IBS-M may instead have functional constipation, whose recognition and treatment would induce resolution of symptoms without performing the stressful, expensive investigations implemented for patients with IBS-D and IBS-M.

As secondary aim we analyzed the costs for investigation and treatment sustained by the families in the last year before referral to our Unit compared with the costs of our diagnostic work-up.

## Methods

Between January and July 2018, 26 children were consecutively referred to our outpatient pediatric gastroenterology clinic having previously been diagnosed with IBS, allegedly according to Rome IV criteria: 10 IBS-D and 16 IBS-M*.* Diagnostic criteria for IBS subtypes were, in brief, the following [8]: IBS-D, that is with predominant diarrhea, more than one-fourth (25%) of bowel movements with Bristol stool form types 6 or 7 and less than one fourth (25%) of bowel movements with Bristol stool form types 1 or 2; IBS-M, that is with mixed bowel habits, more than one-fourth (25%) of bowel movements with Bristol stool form types 1 or 2 and more than one fourth (25%) of bowel movements with Bristol stool form types 6 or 7; IBS-C, that is with predominant constipation, more than one-fourth (25%) of bowel movements with Bristol stool form types 1 or 2 and less than one fourth (25%) of bowel movements with Bristol stool form types 6 or 7. These patients had previously undergone, in other hospitals, a diagnostic work-up that ruled out organic causes such as celiac disease, inflammatory bowel disease and surgically-treatable abdominal conditions. After approval of the ethics committee of the University of Messina Hospital “Policlinico G. Martino” and parents’ verbal informed consent, we enrolled the children in a 2-month prospective study aimed at verifying the hypothesis that they had occult constipation only. For the purpose of this investigation, we chose to define occult constipation when at least one of the following elements was present: 1) a history of FC in the first 3 years of life, 2) palpable fecal masses on physical exam, 3) soiling without other Rome IV criteria of FC, 4) excessive fecal load on plain abdominal radiograph performed if excessive fecal burden was suspected in patients whose physical examination was unreliable/not possible [2]. Our diagnostic work-up simply included IgA anti-tissue transglutaminase antibodies (tTG-IgA) in all the patients with IBS-D and IBS-M, due to the presence of diarrhea, in order to rule out celiac disease. The patients with normal tTG-IgA who fulfilled these criteria were treated for fecal impaction according to evidence-based recommendations [9], starting with a dose of polyethylene glycol (1–1.5 g/Kg per day for 3–6 days) followed by a maintenance dose. At 2 months they were re-evaluated. A group of 16 IBS-C, for whom a treatment for constipation is recommended by Roma IV criteria, were consecutively seen in the same period at the outpatient clinic, considered as control and evaluated after 2 months of treatment. The endpoints at this time were: a decrease of more than 50% of abdominal pain intensity and frequency scores [8] for all, and disappearance of diarrhea as result of treating constipation. Pain intensity was scored using the validated 6-face Faces Pain Scale Revised intensity score [10], while pain frequency was scored, according to Korterink et al. [11] as follows: 0 ¼ no daily pain, 1 ¼ 0–20 min of daily pain, 2 ¼ 20–40 min of daily pain, 3 ¼ 40–90 min of daily pain, and 4 ¼ > 90 min of daily pain. At the 2-month control, the maintenance dose was confirmed but it was allowed to stop or to decrease it at choice of the patients, parents and family pediatricians. A follow-up was planned at least 6 months for evaluating the duration of treatment and the outcome with and without treatment.

We analyzed the costs for investigation and treatment sustained by the families in the last year before referral to our Unit by recording them through a structured questionnaire. These estimates were then compared with the costs of our diagnostic work-up.

### Statistical analysis

The differences of response of IBS-D and IBD-M groups compared with IBS-C group were estimated with the Fisher’s exact test.

## Results

Demographic data, occult constipation signs in IBS-D and IBS-M groups, response to treatment, costs and drop out in the 3 subtypes of patients with IBS are shown in the Table 1.

**Table 1:** Demographic data, response to treatment and costs in patients initially diagnosed as having IBS

IBS subtypes *n* = 42	Mean age (range)	Gender		Occult constipationsigns *n* = 40	Response = Occult constipation	No Response =IBS	Average Costs per person (range)	Potential cost saving (% with a positive diagnosis)
		M	F					
IBS-D *n* = 10	13,5 yrs. (9.5–18)	9	1	a) *n* = 2 b) *n* = 4 c) *n* = 5	8 (80%)	1 (+ 1 dropout)	€ 341.9 (0–996,89)	87%
IBS-M *n* = 16	11 yrs. (5–16.2)	9	7	a) *n* = 5 b) *n* = 14 c) *n* = 10	14 (87%)	0 (+ 2 dropout)	€ 191.15 (0–585.03)	76,78%
IBS-C *n* = 16	9,2 yrs. (5.1–14.8)	5	11	**/**	15 (93%)	1	€ 167.08 (0–508.59)	73,44%

a) history of FC during the first three years of life; b) presence of palpable fecal masses on abdominal exam; c) soiling in the absence of other Rome IV criteria of FC.

The response was not significantly different at *p* < 0.05, from that observed in 15 (93%) of the IBS-C control group (Fisher exact test statistical value 0.5 and 0.6, respectively).

Out of the 2 dropouts in the IBS-M group, one who presented response at 2 months was excluded because she developed an autoimmune cholangitis and thus stopped the follow-up. “Diarrhea” subsided after the first week of treatment in 8 of the 10 IBS-D patients and in all of 14 IBS-M patients. With regard to abdominal pain: a response was observed in all patients across the 3 IBS groups, with the single exception of 1 IBS-C child. Of note: only 2 of the 42 IBS children*,* excluding 2 patients who dropped out, were eventually classified as true IBS, having shown no response to our treatment for constipation. The mean (SD) duration of treatment after the first evaluation at 2 months was 5.5 months ±3.7 (range 1–12).

The mean duration of follow-up was 10.1 months ±2.03 (range 6–13 months). Four IBS-D and 8 IBS-M patients discontinued treatment after 3–11 months and had resolution of symptoms at the follow-up (range 8–14 months)*.* Four patients with IBS-D and 6 patients with IBS-M who discontinued treatment after 40 days-12 months, had a recurrence of symptoms (abdominal pain, diarrhea or constipation). They resumed treatment and had resolution of symptoms at the follow-up (range 6-15 months).

tTG-IgA titers were normal in all the patients with IBS-D and IBS-M. Plain abdominal radiographs were not performed in any case. Potential cost saving of applying our approach, including both tTG IgA and plain abdominal radiograph would have been 73.4% for the 15 patients with IBS-C; 87% for the 8 IBS-D patients and 76.8% for the 14 IBS-M patients. An overall saving of 79.26% was estimated for the whole group of “IBS” patients.

## Discussion

Our preliminary results in a small series of children previously thought to have IBS-D or IBS-M suggest that many patients receiving this diagnosis have in fact functional constipation. Indeed, we showed a response of abdominal pain to constipation treatment not only in patients with IBS-C, as may be expected according to Rome IV criteria, but also in those that otherwise would be considered as having IBS-D and IBS-M. Moreover “diarrhea”, evidently due to soft or liquid stools overflowing around the large fecal mass [12], completely regressed.Therefore, we suggest that, when clinical signs of occult constipation as we defined them are present, treatment for constipation should be offered not only in IBS-C as suggested by Rome IV criteria, but also in patients thought to be possibly IBS-D or IBS-M. Subjects who respond to treatment should be considered as having functional constipation only. Despite being such a common clinical problem, chronic constipation is underestimated [13]. Indeed, in our study only one child was referred to the outpatient clinic of pediatric gastroenterology for constipation. In IBS-D and IBS-M, soiling due to fecal incontinence was considered by parents as a consequence of diarrhea. The presence of fecal incontinence alone does not meet Rome IV diagnostic criteria for FC [2], but we considered it a sign of occult constipation (present in 15 out of 23 patients with IBS-D or IBS-M, see Table 1). A recent study in the Netherlands [14] assessed the costs of diagnostic evaluation of chronic abdominal pain in children diagnosed with irritable bowel syndrome or functional abdominal pain. Total annual medical and non-medical costs of care were estimated to be more than € 2500 per patient.The present study has estimated mean costs per person suffering from IBS to be €214, with IBS-D being the most expensive as its diagnostic workup is aimed at ruling out inflammatory bowel diseases. We estimated that by performing determination of tTG-IgA and plain abdominal radiograph we could achieve a 79.26% reduction of costs that may be even greater considering that in our study we had no indication for the abdominal X-ray.

Limitations to our study should be taken into account.

Firstly, ours was an observational, uncontrolled study, as our aim was to preliminarily verify our hypothesis as a proof of concept trial calling for future controlled studies. However, in the follow up the reappearance of abdominal pain after stopping treatment and disappearance after resuming it strongly suggests a cause/effect relationship between constipation and symptoms.

Secondly, placebo effect, a well-known occurrence in FGID, might have been contributing to the symptoms’ resolution. However, according to what proposed by Gjilsberg et al. [4] we reduced the potential placebo effect by requiring persistence of the therapeutic effect during a follow-up period of at least 6 months, a time considered adequate to dissipate any placebo effect [15].

Furthermore, a placebo effect seems less probable on “diarrhea” that subsided in our IBS patients with treatment of constipation, the main novelty of our study.

## Conclusions

While our preliminary results should be confirmed by controlled studies, we suggest that an empirical treatment for constipation should be undertaken in presence of apparent IBS when signs of occult constipation are present. As we have shown, such approach would also have the added value of resulting in significant cost savings. Education and awareness amongst parents and nursing staff is important to differentiate functional constipation from IBS, as often the diagnosis of IBS can cause extreme anxiety to caregivers/parents of children.

## Data Availability

The datasets used and/or analyzed during the current study are available from the corresponding author on reasonable request.

## Abbreviations

FC: Functional constipation
FGIDs: Functional gastrointestinal disorders
IBS: Irritable bowel syndrome
IBS-C: Irritable bowel syndrome with constipation
IBS-D: Irritable bowel syndrome with diarrhea
IBS-M: Irritable bowel syndrome mixed
tTG-IgA: IgA anti-tissue transglutaminase antibodies
